# Fine-tuning Flowering Time via Genome Editing of Upstream Open Reading Frames of Heading Date 2 in Rice

**DOI:** 10.1186/s12284-021-00504-w

**Published:** 2021-06-29

**Authors:** Xinxin Liu, Hualong Liu, Yuanye Zhang, Mingliang He, Rongtian Li, Wei Meng, Zhenyu Wang, Xiufeng Li, Qingyun Bu

**Affiliations:** 1 College of Life Science, Northeast Forestry University, Harbin, 150040 China; 2 Northeast Institute of Geography and Agroecology, Key Laboratory of Soybean Molecular Design Breeding, Chinese Academy of Sciences, Harbin, 150081 China; 3 Rice Research Institute, College of Agriculture, Northeast Agricultural University, Harbin, 150030 China; 4 College of Life Science, Heilongjiang University, Harbin, 150080 China; 5 Graduate University of Chinese Academy of Sciences, Beijing, 100049 China

**Keywords:** Rice, Heading date, Upstream open reading frames, CRISPR/Cas9

## Abstract

Flowering time of rice (*Oryza sativa L.*) is among the most important agronomic traits for region adaptation and grain yield. In the process of rice breeding, efficient and slightly modulating the flowering time of an elite cultivar would be more popular with breeder. Hence, we are interested in slightly increasing the expression of flowering repressors by CRISPR/Cas9 genome editing system. It was predicated there were three uORFs in 5’ leader sequence of *Hd2*. In this study, through editing *Hd2* uORFs, we got four homozygous mutant lines. Phenotypic analysis showed that the *hd2 urf* edited lines flowered later by 4.6–11.2 days relative to wild type SJ2. Supporting the later flowering phenotype, the expression of *Ehd1*, *Hd3a*, and *RFT1* is significantly decreased in *hd2 urf* than that in wild type. Moreover, we found that the transcription level of *Hd2* is not affected, whereas the Hd2 protein level was increased in *hd2 urf* compared with wild type, which indicated that Hd2 uORFs indeed affect the translation of a downstream Hd2 pORF. In summary, we developed a efficient approach for delaying rice heading date based on editing uORF region of flowering repressor, which is time and labor saving compared to traditional breeding. In future, uORF of other flowering time related genes, including flowering promoter and flowering repressor genes, can also be used as targets to fine-tune the flowering time of varieties.

**Supplementary Information:**

The online version contains supplementary material available at 10.1186/s12284-021-00504-w.

Rice, one of the world’s most important cereal crops, is a staple food for more than half of all humans alive. Rice-growing regions range across a large part of the Earth, from 40°S to 53°N. To adapt to local circumstances, rice plants need to flower and ripen at the correct time. Flowering time, also called heading date, is one of the most important agronomic traits in rice. It determines rice distribution and final yield. Flowering time is a very complicated trait, and it is controlled by multiple internal genetic factors and many different external environmental factors (Hori et al. 2016; Cho et al. 2017). Over the past few decades, extensive molecular genetics studies have identified numerous genes involved heading date in rice (Hori et al. 2016). Studies have shown that various combinations of heading-date-related genes with multiple natural variations are the final determinants of local adaptability of a specific cultivar (Gao et al. 2014; Li et al. 2015; Zhang et al. 2015; Fujino et al. 2019).

Rice is a short-day (SD) plant, and the flowering time of rice is very sensitive to day length (Hori et al. 2016). Short-day conditions promote flowering, and long-day (LD) conditions delay flowering (Hori et al. 2016). The photoperiod pathway is the most important flowering time regulatory pathway in rice (Song et al. 2013). Rice has two major photoperiod-dependent flowering pathways, an evolutionarily conserved OsGI-Hd1-Hd3a pathway and a specific Ehd1-Hd3a pathway (Hori et al. 2016). Ehd1 activates the expression of two florigens, Heading date 3a (Hd3a) and RICE FLOWERING LOCUS T1 (RFT1), to promote flowering (Doi et al. 2004).

Both Hd1 and Ehd1 are regulated by many different flowering time regulators that modulate the transcription or protein level of Hd1 and Ehd1 (Hori et al. 2016). In Hd1-dependent pathway, Hd1 is an activator under SD conditions, while it acts as a suppressor under LD (Yano et al. 2000). Hd1 protein can be phosphorylated by OsK4 and ubiquitinated by HAF1 at the post-translation level (Yang et al. 2015; Sun et al. 2016). In the Ehd1-dependent pathway, Ehd1 is regulated by multiple suppressors (e.g., Hd2, Hd4, Hd5, OsCOL4, OsCOL10, OsLFL1) and activators (e.g., DTH3, OsMADS51, OsMADS56, Ehd2, Ehd4) (Hori et al. 2016).

In the process of rice breeding, efficient, slight modulation of flowering time of an elite cultivar is very popular with breeders. The CRISPR/Cas9 genome editing system has been proven to be a suitable technique for breeding rice, and a number of major traits have been edited successfully so far (Mao et al. 2019). Previous studies have shortened the rice flowering time by editing many types of flowering repressor genes (Li et al. 2017; Cui et al. 2019). However, it is still necessary to delay the flowering time in rice breeding. There have been two ways to meet this demand, increasing the expression of the flowering repressor and decreasing the expression of the flowering promoter. The rice flowering regulatory pathway has fewer flowering promoter genes than flowering repressor genes (Hori et al. 2016). In addition, the effect of flowering promoter genes (e.g., *Ehd1*, *Hd3a*, and *RFT1*) is too strong for them to be used as target genes of editing because the loss of function of *Ehd1*, *Hd3a*, and *RFT1* can strikingly delay flowering or prevent it entirely, which limits their practical breeding values (Zhao et al. 2015).

Hence, we attempted to slightly increase the expression of a flowering repressor, and mildly delay flowering in rice. It has been reported that upstream open reading frames (uORFs) can repress the translation of downstream primary ORFs (pORFs) and that the protein level of pORFs can be increased via editing and mutating the uORFs (Zhang et al. 2018). Following this logic, we sought to search and mutate the uORFs in the flowering repressor genes via genome editing. We performed an online search of the plant uORFs database (http://uorflight.whu.edu.cn/) (Niu et al. 2020). Our findings indicated that there may exist three uORFs in 5′ leader sequence of *Hd2*. These uORFs were named uORF 1, uORF 2, and uORF 3. We chose these as targets for genome editing (Fig. 1A).

**Fig. 1:**
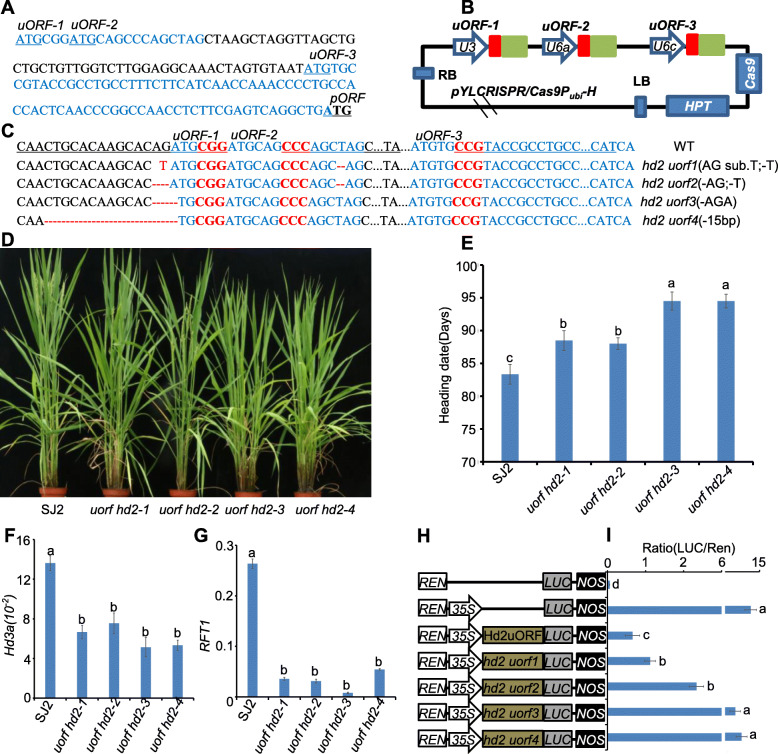
Editing of *Hd2* uORFs delays rice heading date. **A** There are three uORFs in 5’ leader sequence(blue) of *Hd2*, and uORFs putative initiation codon is underlined.pORF is shown in bold with underlined. **B **Schematic diagram of the CRISPR/Cas9 vector. Three target sequences (*uORF-1,uORF-2,uORF-3*) fused with sgRNA scaffolds were driven by respective *U3* or *U6* promoters. The three sgRNA expression cassettes were sequentially inserted into the binary vector *pYLCRISPR/Cas9P*_*ubi*_-*H*. Red boxes indicate three target sequences and green boxes indicate sgRNA scaffolds. *HPT*, hygromycin phosphotransferase gene. **C** Four homozygous mutants (T_2_ generation) of *hd2 uorf1* to *hd2 uorf4* obtained by CRISPR/Cas9 editing. The uORF sequence (blue) is shown with the sgRNA target site underlined and the protospacer-adjacent motif shown in red. The nucleotide changes are labeled in red. ‘-’ means deletion and sub. means substitution. **D** Representative flowering image of mutants *uorf hd2-1* to *uorf hd2-4* indicated genotypes under NLD in summer at Harbin. Wide type SJ2 was used as control. **E** Flowering time of each genotype under NLD conditions. Data are means ± SE (*n* = 20). **F** and **G** qRT-PCR analysis of *Hd3a* (**F**) and *RFT1* (**G**) transcription level in indicated lines and SJ2. Rice *UBIQUITIN* gene was used as the internal control. Means and standard deviations were obtained from three biological replicates. Data are means ± SE (*n* = 3). **H **Schematic diagrams of the reporter plasmids used in rice protoplasts transient assay. REN, Renilla luciferase; LUC, firefly luciferase. **I** The LUC activity in rice protoplasts with indicated reporter plasmids. Data are means±SE (*n*=3). Statistically significant differences are indicated by different lowercase letters (*P* < 0.05, one-way ANOVA with Tukey’s significant difference test)

First, we designed three sgRNAs to target uORF 1, uORF 2, and uORF 3 (Fig. 1B). These sgRNA catastases were sequentially ligated into the CRISPR/Cas9 binary vectors p*YLCRISPR/Cas9P*_*ubi*_*-H* (Ma et al. 2015). Songjing 2 (SJ2), an elite rice variety grown in Heilongjiang Province, was chosen for transformation. At the T_0_ generation, we produced 12 independent transgenic lines (Fig. S1). In the T_1_ generation, the mutations uORF 1, uORF 2, and uORF 3 in these lines were determined through sequencing (Table S1). Results showed that, in lines 1–4, the mutation type of editing target is homozygous, and they were named *uorf hd2-1* to *uorf hd2-4* for further investigation (Fig. 1C). In contrast, lines 5–8, with heterozygous mutation, and lines 9–12, which were not edited in the target, were not studied further (Fig. S2). Moreover, we found that uORF 1 was edited in *uorf hd2-1* to *uorf hd2-4*, uORF 2 was edited in lines *uorf hd2-1* and *uorf hd2-2*, and uORF 3 was not edited in four lines (Fig. 1C). Notably, the sites of mutation for the uORF 1 target were very close to ATG, and initiation codon of uORF1 was deleted in *uorf hd2-3* and *uorf hd2-4* (Fig. 1C). These results also indicated that the target of uORF 1 might be easier to edit than those of uORF 2 and uORF 3.

At the T_2_ generation, four homozygous lines *uorf hd2-1* to *uorf hd2-4* were investigated in detail. Phenotypic analysis showed that the *uorf hd2-1* to *uorf hd2-4* transformation delayed flowering by 4.6–11.2 days relative to wild-type SJ2, and *uorf hd2-3* and *uorf hd2-4* delayed flowering to a greater extent than *uorf hd2-1* and *uorf hd2-2* did (Fig. 1D, E). Supporting this result, qRT-PCR analysis showed the expression of florigen genes *Hd3a* and *RFT* to be significantly lower in *uorf hd2-1* to *uorf hd2-4* than in SJ2 (Fig. 1F,G). Like those of *Hd3a* and *RFT*, *Ehd1* levels were also markedly lower in *uorf hd2-1* to *uorf hd2-4* mutants than in SJ2 (Fig. S3). We also examined the expression of *Hd2* and found the *Hd2* transcription level to be comparable between SJ2 and four edited lines. These results indicated that the mutated uORFs of *Hd2* do not affect the transcription of pORF of *Hd2*, which is consistent with previous reports (Zhang et al. 2018). It has been shown that uORF performs its function by suppressing the translation efficiency and protein level of pORF (Zhang et al. 2018). We thus attempted to examine the protein level of Hd2 in *uorf hd2-1* to *uorf hd2-4*. Because of loss of effective Hd2 antibody, we could not directly detect the Hd2 protein level rice plants. To overcome this obstacle, we used the rice protoplast system combined with the dual-luciferase reporter system to determine whether mutated Hd2 uORF can affect translation of a downstream Hd2 pORF. As shown in Fig. 1H, five constructs were made in which wild-type *Hd2 uORF* and four mutated *hd2 uorf* (mutated *hd2 uorf* in *uorf hd2-1* to *uorf hd2-4*, respectively) were inserted between the *35 S* promoter and LUC reporter. The translation level of LUC was recorded by reading the ratio of LUC and REN. Results showed that the expression of LUC directed by *35 S* promoter to be very high, and the insertion of *Hd2 uORF* largely suppressed the expression of LUC (Fig. 1I). However, when the *Hd2 uORF* is replaced by *hd2 uorf*, the expression of LUC recovered to different significant extents, indicating that the *Hd2 uORF* can indeed suppress the expression *Hd2 pORF* (Fig. 1I). The degree of recovery in *hd2 uorf 3* and *hd2 uorf 4* was much larger than that in *hd2 uorf 1* and *hd2 uorf 2* (Fig. 1I). Simultaneously, we found the transcription levels of LUC normalized to the REN were comparable between *Hd2 uORF* and four *hd2 uorf*, which indicated that *Hd2 uORF* could indeed suppress the translation but not transcription of *Hd2 pORF*. (Fig. S4). In addition, the differential LUC expression caused by different *hd2 uorf* was found to be consistent with a delaying flowering phenotype in *uorf hd2-1* to *uorf hd2-4* (Fig. 1E), suggesting that *Hd2 uORF* is an efficient target for generating later flowering rice cultivars.

Statistically significant differences are indicated by different lowercase letters (*P* < 0.05, one-way ANOVA with Tukey’s significant difference test).

In summary, we developed an efficient approach to delaying rice heading date based on editing uORF region of the flowering repressor, which saves time and labor over traditional breeding. In the future, the uORF of other flowering-time-related genes, including flowering promoter and flowering repressor genes, can also be used as targets to fine-tune the flowering time of rice varieties.

## Supplementary Information

**Additional file 1: Supplemental Figure 1.** Identification of transgenic plants in T_0_ generation. **Supplemental Figure 2.** Four heterozygous mutants of *hd2 uorf5* to *hd2 uorf8* obtained by CRISPR/Cas9 editing. **Supplemental Figure 3.** qRT-PCR analysis of *Hd2* and *Ehd1* transcription level in indicated lines and SJ2. **Supplemental Figure 4.** qRT-PCR analysis of *LUC* transcription level in rice protoplast system. **Supplemental Table 1.** Editing efficiency analysis in T_1_ generation. **Supplemental Table 2.** Primers used in this study.

## Data Availability

All data generated or analyzed during this study are included in this published article and its supplementary information files.

## Abbreviations

SD: Short-day
LD: Long day
GI: GIGANTEA
Hd1: Heading date1
Ehd1: Early heading date1
Hd3a: Heading date 3a
RFT1: RICE FLOWERING LOCUS T1
HAF1: Heading date Associated Factor 1
Hd2: Heading date 2
Hd4: Heading date 4
Hd5: Heading date 5
COL4: CONSTANS-like 4
COL10: CONSTANS-like 10
OsLFL1: LEC2 and FUSCA3 like 1
DTH3: Days to Heading 3
Ehd2: Early Heading Date 2
Ehd4: Early Heading Date 4
CRISPR: Clustered regulatory interspaced short palindromic repeat
uORFs: Upstream Open Reading Frames
pORF: Primary Open Reading Frame
LUC: Firefly luciferase
REN: Renilla luciferase
