# Correction to: Fine-tuning Flowering Time via Genome Editing of Upstream Open Reading Frames of Heading Date 2 in Rice

**DOI:** 10.1186/s12284-021-00511-x

**Published:** 2021-07-20

**Authors:** Xinxin Liu, Hualong Liu, Yuanye Zhang, Mingliang He, Rongtian Li, Wei Meng, Zhenyu Wang, Xiufeng Li, Qingyun Bu

**Affiliations:** 1 College of Life Science, Northeast Forestry University, Harbin, 150040 China; 2 Key Laboratory of Soybean Molecular Design Breeding, Northeast Institute of Geography and Agroecology, Chinese Academy of Sciences, Harbin, 150081 China; 3 Rice Research Institute, College of Agriculture, Northeast Agricultural University, Harbin, 150030 China; 4 College of Life Science, Heilongjiang University, Harbin, 150080 China; 5 Graduate University of Chinese Academy of Sciences, Beijing, 100049 China

**Correction to: Rice 14, 59 (2021)**

**https://doi.org/10.1186/s12284-021-00504-w**

It was highlighted that in the original article (Liu et al. 2021) the affiliations were incorrect and presented as below. The original article has been updated.

**Incorrect Affiliations**

Xinxin Liu1†, Hualong Liu2†, Yuanye Zhang3†, Mingliang He4,5, Rongtian Li3, Wei Meng1, Zhenyu Wang4, Xiufeng Li4* and Qingyun Bu4*

1College of Life Science, Northeast Forestry University, 150081 Harbin, China.

2College of Life Science, Heilongjiang University, 150080 Harbin, China.

3Northeast Institute of Geography and Agroecology, Key Laboratory of Soybean Molecular Design Breeding, Chinese Academy of Sciences, 150081 Harbin, China.

4Rice Research Institute, College of Agriculture, Northeast Agricultural University, 150030 Harbin, China.

5University of Chinese Academy of Sciences, Beijing 100049, China.
