# Supplementary material for: Fine-tuning Flowering Time via Genome Editing of Upstream Open Reading Frames of Heading Date 2 in Rice
Source: Rice (N Y). 2021 Jun 29;14:59. doi: 10.1186/s12284-021-00504-w (PMC8241947; doi:10.1186/s12284-021-00504-w)
Supplement: Supplementary file 1 — Additional file 1: Supplemental Figure 1. Identification of transgenic plants in T0 generation. Supplemental Figure 2. Four heterozygous mutants of hd2 uorf5 to hd2 uorf8 obtained by CRISPR/Cas9 editing. Supplemental Figure 3. qRT-PCR analysis of Hd2 and Ehd1 transcription level in indicated lines and SJ2. Supplemental Figure 4. qRT-PCR analysis of LUC transcription level in rice protoplast system. Supplemental Table 1. Editing efficiency analysis in T1 generation. Supplemental Table 2. Primers used in this study. [file 12284_2021_504_MOESM1_ESM.docx]

**Supplemental data**

**Supplemental Figure 1. Identification of transgenic plants in T_0_ generation**

M indicates DL2000 DNA Ladder Mix; Line 1-13 are transgenic plants; Line 14 is water used as a negative control; Line 15 is WT SJ2.

**Supplemental Figure 2. Four heterozygous mutants of *hd2 uorf5* to *hd2 uorf8* obtained by CRISPR/Cas9 editing.**

The uORF sequence is shown with red arrows.

**Supplemental Figure 3. qRT-PCR analysis of *Hd2* and *Ehd1* transcription level in indicated lines and SJ2.**

Rice *UBIQUITIN* gene was used as the internal control. Means and standard deviations were obtained from three biological replicates. Data are means±SE (n=3).

**Supplemental Figure 4. qRT-PCR analysis of *LUC* transcription level in rice protoplast system.**

The relative *LUC* transcription level normalized to the *REN* transcription level are shown (LUC/REN, n = 3). Rice *UBIQUITIN* gene was used as the internal control. Means and standard deviations were obtained from three biological replicates. Data are means±SE (n=3).

**Supplemental Table 1. Editing efficiency analysis in T1 generation.**

|  | 1 | 2 | 3 | 4 | 5 | 6 | 7 | 8 | 9 | 10 | 11 | 12 |
| --- | --- | --- | --- | --- | --- | --- | --- | --- | --- | --- | --- | --- |
| uORF1 | H | H | H | H | Z | Z | Z | Z | W | W | W | W |
| uORF2 | H | H | W | W | Z | Z | Z | Z | W | W | W | W |
| uORF3 | W | W | W | W | Z | Z | Z | Z | W | W | W | W |

Note: Z means heterozygosis, H means homozygosis, W means WT.

**Supplemental Table 2. Primers used in this study.**

| **Primer name** | **Primer sequence (5'-3')** |
| --- | --- |
| U3-uORF1-LP | GGCACAACTGCACAAGCACAGATG |
| U3-uORF1-RP | AAACCATCTGTGCTTGTGCAGTTG |
| U6a-uORF2-LP | GCCGTAACCTAGCTTAGCTAGCT |
| U6a-uORF2-RP | AAACAGCTAGCTAAGCTAGGTTA |
| U6c-uORF3-LP | TCAGATGAAGAAAGGCAGGCGGTA |
| U6c-uORF3-RP | AAACTACCGCCTGCCTTTCTTCAT |
| Sequencing-F | CCGCCCTCCCCAATATCTAG |
| Sequencing-R | TGGTTGTGATGAGCGGTTCC |
| HPT-F | TGCGCCCAAGCTGCATCAT |
| HPT-R | TGAACTCACCGCGACGTCTGT |
| QRT-Hd3a-F | GCTCACTATCATCATCCAGCATG |
| QRT-Hd3a-R | CCTTGCTCAGCTATTTAATTGCATAA |
| QRT-RFT1-F | TGACCTAGATTCAAAGTCTAATCCTT |
| QRT-RFT1-R | TGCCGGCCATGTCAAATTAATAAC |
| QRT-Ehd1-F | ATGGCTTCAAGTGGAGACAC |
| QRT-Ehd1-R | ATATTGATGGAGGATGACCG |
| QRT-Hd2-F | TCTGGAGGAGTCAAGTGTTCG |
| QRT-Hd2-R | TTTCCCTTCTGGCACTTTGG |
| QRT-LUC-F | ATTACCAGGGATTTCAGTCG |
| QRT-LUC-R | CAATTGTCTTGTCCCTATCG |
| QRT-REN-F | GCCTCGTGAAATCCCGTTAG |
| QRT-REN-R | TTGGCACCTTCAACAATAGC |
| pGreen-35S-F | GGGCCCCCCCTCGAGGTCGACACGTTGTAAAACGACGGCC |
| pGreen-35S-R | CGCTCTAGAACTAGTGGATCCACGGAGCAAGGGGAGAGGGAG |
| pGreen-35S-uORFHd2-F | CTCCCTCTCCCCTTGCTCCGTGGATCCTTTGCACAACTGCACAAGCA |
| pGreen-35S-uORFHd2-R | CGCTCTAGAACTAGTGGATCC CAGCCTGACTCGAAGAGGTTG |

**Materials and methods**

**Rice materials and growth conditions**

Songjing 2 (*Oryza sativa*) cultivars was used for *Agrobacterium*-mediated co-cultivation transformation experiments, as described previously (Tian et al., 2015). Plants were grown in Harbin (45°N) with natural long day condition in the paddy field (Li et al., 2015). Heading dates were recorded from sowing to the appearance of the first panicle (Li et al., 2015).

**Plasmid construction**

It was predicated there were three uORFs in 5' leader sequence of *Hd2*. The target sequence near the uORFs were selected and target sequence-containing chimeric primers were designed (Supplemental Table 2), and ligated into the CRISPR/Cas9 binary vectors p*YLCRISPR/Cas9P_ubi_-H* (Ma et al., 2015).

**Rice Transformation and Mutation Detection**

The CRISPR/Cas9 constructs were introduced into *Agrobacterium tumefaciens* strain EHA105 by heat-shock. Rice transformation was performed as described previously (Tian et al., 2015)*.* At T_0_ generation, genomic DNA was extracted from leaves of transgenic rice plants. PCR amplifications were carried out using hygromysin gene specific primer to identify the positive transgenic plants (Supplemental Table 2). the uORFs region was then amplified with sequencing primers and sequenced, the transgenic lines with mutation were chosen and used for further analysis.

**Gene expression analysis**

Total RNA was extracted using TRIzol (Invitrogen). cDNA was synthesized from 1.5 µg of total RNA using PrimeScript™ 1st Strand cDNA Synthesis Kit (Takara). Real-time PCR was performed with SYBR Green PCR master mix (TransGen). Data were collected using LightCycler480 II real-time PCR detector. All expressions were normalized against the *ubiquitin* gene (Os01g0328400). The primers used are listed at Supplemental Table 2. Three biological repeats were performed for each analysis.

**Protoplast transient transformation assays**

The *pGreenII 0800-LUC* empty vector was used as negative control, and *35S* promoter was cloned into *pGreenII 0800-LUC* vector used as positive control, (Supplemental Table 2). The uORF of *Hd2* and four mutated *hd2* were inserted between 35S promoter and LUC reporter of pGreenII 0800-LUC vector. The Renilla luciferase (REN) gene directed by *35S* promoter was used as an internal control. Firefly LUC and REN activities were measured with a Dual-Luciferase reporter assay kit (Beyotime, RG027) using a GloMax 20/20 luminometer (Promega). The LUC activity was normalized to REN activity and LUC/REN ratios were calculated. For each plasmid, three independent transformations were performed. Values are means ± SE of three biological repeats.

Li X F, Liu H Z, Wang M Q, Liu H L, Tian X J, Zhou W J, Lv T X, Wang Z Y, Chu C C, Fang J, Bu Q Y. 2015. Combinations of Hd2 and Hd4 genes determine rice adaptability to Heilongjiang Province, northern limit of China. *Journal of integrative plant biology*, **57**(8): 698-707.

Ma X L, Zhang Q Y, Zhu Q Y, Liu W, Chen Y, Qiu R, Wang B, Yang Z F, Li H Y, Lin Y R, Xie Y Y, Shen R X, Chen S F, Wang Z, Chen Y L, Guo J X, Chen L T, Zhao X C, Dong Z C, Liu Y G. 2015. A robust CRISPR/Cas9 system for convenient, high- efficiency multiplex genome editing in monocot and dicot plants. *Mol Plant*, **8**(8): 1274–1284.

Tian X J, Wang Z Y, Li X F, Lv T X, Liu H Z, Wang L Z, Niu H B, Bu Q Y. 2015. Characterization and Functional Analysis of Pyrabactin Resistance-Like Abscisic Acid Receptor Family in Rice. *Rice*, **28**(8): 1-13.
